# Negative Frequency-Dependent Selection Is Frequently Confounding

**DOI:** 10.3389/fevo.2018.00010

**Published:** 2018-02-21

**Authors:** Dustin Brisson

**Affiliations:** Biology Department, University of Pennsylvania, Philadelphia, PA, United States

**Keywords:** negative frequency dependent selection, balancing selection, killing the winner hypothesis, multiple niche polymorphism, density dependent selection

## Abstract

Persistent genetic variation within populations presents an evolutionary problem, as natural selection and genetic drift tend to erode genetic diversity. Models of balancing selection were developed to account for the maintenance of genetic variation observed in natural populations. Negative frequency-dependent selection is a powerful type of balancing selection that maintains many natural polymorphisms, but it is also commonly misinterpreted. This review aims to clarify the processes underlying negative frequency-dependent selection, describe classes of polymorphisms that can and cannot result from these processes, and discuss the empirical data needed to accurately identify processes that generate or maintain diversity in nature. Finally, the importance of accurately describing the processes affecting genetic diversity within populations as it relates to research progress is considered.

## INTRODUCTION

Natural diversity—the “endless forms most beautiful and most wonderful” (Darwin, 2012)—Is an enduring focus of both evolutionary biologists and nature lovers. The evolutionary processes that have generated or are maintaining many examples of diversity in nature, however, remain obscure and often controversial (Chesson, 2000). The processes that result in persistent polymorphisms within populations demand a special explanation as both directional natural selection and genetic drift should eliminate alleles and thus erode genetic diversity (Lewontin, 1974; Charlesworth and Hughes, 2000; Nielsen, 2005). Nevertheless, many examples of persistent polymorphisms occur in nature (Hedrick, 1986; Mallet and Joron, 1999; Richman, 2000; Carius et al., 2001; Hedrick et al., 2002; Delph and Kelly, 2014). Models of balancing selection—including negative frequency-dependent selection, spatial or temporal habitat heterogeneity, and heterozygote advantage—provide theoretical frameworks describing the processes that can account for persistent polymorphisms within populations. A core tenet of each balancing selection model is that the selective value of an allele—whether it is beneficial or detrimental—is dependent on the environmental context (Dobzhansky, 1982; Clarke et al., 1988). That is, alleles are advantageous and deleterious in different circumstances.

Negative frequency-dependent selection has been called the most powerful selective force maintaining balanced polymorphisms (Ayala and Campbell, 1974; Turelli and Barton, 2004; Fitzpatrick et al., 2007; Kazancioǧlu and Arnqvist, 2014), with some proposing that a large proportion of natural genetic polymorphisms are maintained by selection favoring rare alleles (Kojima and Yarbrough, 1967). Negative frequency-dependent selection occurs when the selective value of a variant (relative to other variants) is a function of its abundance in the population (relative to other variants) such that its relative fitness increases as the relative abundance, or frequency, of the variant decreases (Wright, 1939) (please see Clarke, 1979; Levin, 1988 for foundational mathematical descriptions and assumptions of this process). That is, rare variants have a selective advantage specifically because of their rarity while common variants are disadvantaged because of their commonness. Negative frequency-dependent selection has the potential to maintain polymorphisms within populations because relatively rare variants have a selective advantage over more common variants and thus tend to increase in frequency and avoid local extinction. Negative frequency-dependent selection models are a narrow subset of a broad field of models describing the impact of variant frequency on natural selection; the overwhelming majority of this broad field is beyond the scope of the concepts addressed here. Here, I focus on natural polymorphisms that can be explained by negative frequency-dependent selection, where genetic diversity is maintained when a variant becomes disadvantageous as it becomes more frequent, and polymorphisms that are more accurately explained by other process.

Numerous ecological interactions can result in a selective advantage for relatively rare alleles including sexual selection, parasite or predator preferences, and resource competition. In fact, each of these mechanisms has been shown to create a selective advantage for rare alleles that has resulted in persistent polymorphisms in multiple natural populations (Fisher, 1930; Wright, 1939; Harvey et al., 1975; Gigord et al., 2001; Delph and Kelly, 2014). While ecological context and natural history determine the proximate ecological mechanism affecting the differential survival or reproduction of variants in a population, changes in relative survival or reproduction must be negatively correlated with variant frequency for negative frequency-dependent selection to maintain natural polymorphisms. In a classic example, color polymorphisms are maintained in natural populations of *Cepaea nemoralis* snails by negative frequency-dependent selection because their predators, the song thrush (*Turdus philomelos*), form a search image for the most common morph resulting in much greater predation pressure on the common than the rare morph (Harvey et al., 1975; Allen, 1988). The rare morph can increase in frequency due to the relaxed predation pressure until it becomes common, resulting in a search image switch that now targets the new common morph, a process that maintains this polymorphism in *C. nemoralis* populations. Two luminaries in population genetics—R. Fisher and S. Wright—have also demonstrated the power of negative frequency-dependent selection to maintain diversity in natural systems. Wright famously demonstrated that self-incompatibility alleles, a genetic mechanism in plants to prevent inbreeding, are incredibly diverse because pollen containing a rare allele is more likely to find a receptive mate than pollen containing a common allele (Wright, 1964, 1969; Castric and Vekemans, 2004). Thus, plants with rare alleles have a selective advantage (Figure S1 and Appendix 2). Similarly, Fisher’s principle demonstrates that human males and females are equally frequent because, if one sex were more frequent, parents producing the alternate sex would enjoy an advantage resulting in more grandchildren (Fisher, 1930; Edwards, 1998).

The many incontrovertible demonstrations of the power of negative frequency-dependent selection to maintain polymorphisms in nature have led some to suggest that it is a “pervasive” force maintaining natural diversity (Clarke, 1979). The pervasiveness of negative frequency-dependent selection has been further supported by the perception that “nearly every [selective agent] works in a way liable to produce frequency-dependent selection of the kind that favors rare phenotypes and hinders common ones” (Clarke, 1979). Although negative frequency-dependent selection may be a “powerful, perhaps a dominant, factor maintaining genetic diversity” within populations (Clarke, 1979), many natural polymorphisms are maintained by other evolutionary processes (Allison, 1954; Barton, 1986; Smith, 1990; Weatherall, 1997; Schmidt et al., 2000; Brisson and Dykhuizen, 2004). Nevertheless, many natural polymorphisms have been assumed to result from negative frequency-dependent selection even when empirical data from the system are inconsistent with the theoretical framework in which selection favors relatively rare variants. In this essay, I describe several patterns of allele dynamics that are commonly described in the literature as resulting from negative frequency-dependent selection despite data demonstrating that other causative processes. These processes include allelic diversity resulting from directional selection within a changing ecological context, density-dependent population regulation, other models of balancing selection, and aspects of community ecology. I will discuss concepts and experiments that can aid in identifying the processes underlying patterns of allele dynamics and suggest that accurately identifying the evolutionary process underlying natural patterns facilitates the development of hypotheses and future experiments to determine the ecological interactions or molecular mechanisms at the root of the process.

## DIRECTIONAL SELECTION DESCRIBED AS NEGATIVE FREQUENCY-DEPENDENT SELECTION

Conceptually, negative frequency-dependent selection may be the “most intuitively obvious explanation” of polymorphisms in nature (Trotter and Spencer, 2007). However, the original concept becomes ambiguous, complex, and even controversial as a result of differing definitions and applications in both theoretical and empirical work (Heino et al., 1998). Even some of the greatest thinkers in evolutionary biology have used negative frequency-dependent selection to explain scenarios in which the selective values of alleles are independent of their relative abundance. A prominent example comes from an influential essay by JBS Haldane outlining mechanisms by which infectious diseases drive natural selection in metazoans (Haldane, 1949). Although most of these ideas have been “followed profitably” (very profitably indeed), the negative frequency-dependent selection framework described in this essay appears to be one of the few unsound lines of thought. In this framework, Haldane suggested that a host with a rare defensive phenotype has a selective advantage in the face of highly-adapted pathogens, “For just because of its rarity it will be resistant to diseases which attack the majority of its fellows.” That is, the adapted pathogen has evolved mechanisms to overcome the common defensive phenotypes in host populations but cannot overcome the rare defensive phenotypes. Thus, hosts expressing rare but effective defensive phenotypes, or *escape variants*, enjoy a selective advantage over hosts expressing common but exploitable defenses.

The scenario described by Haldane, however, confounds natural selection favoring a specific (*effective*) phenotype in the current environment with a selective advantage resulting from rarity. Haldane’s escape variants have a selective advantage because they cannot be subverted by the pathogen, not because they are rare. Although both rarity and novelty can result in a selective advantage, the novel defensive phenotype maintains its efficacy against the pathogen not because it is rare, but because it is novel. This point can be illustrated by extending this line of thought to allow migration of many individuals expressing a novel and effective defensive phenotype. These migrants would enjoy the same selective advantage over the previously common resident phenotype, regardless of frequency of the novel phenotype in the population immediately following the mass-migration event. The evolutionary dynamics occurring in this framework do not occur because of rare advantage and, in most cases, will not result in a balanced polymorphism. These evolutionary dynamics are more likely the result of directional selection in a continuously changing environment (Levins, 1968; Lande and Shannon, 1996; Orr, 2005; Collins et al., 2007; Bell, 2010). These two processes—negative frequency-dependent selection and selection in a changing environment—can potentially be distinguished by artificially manipulating variant frequencies or by introducing a previously common but now extinct variant into a controlled population.

**FIGURE 1 ∣:**
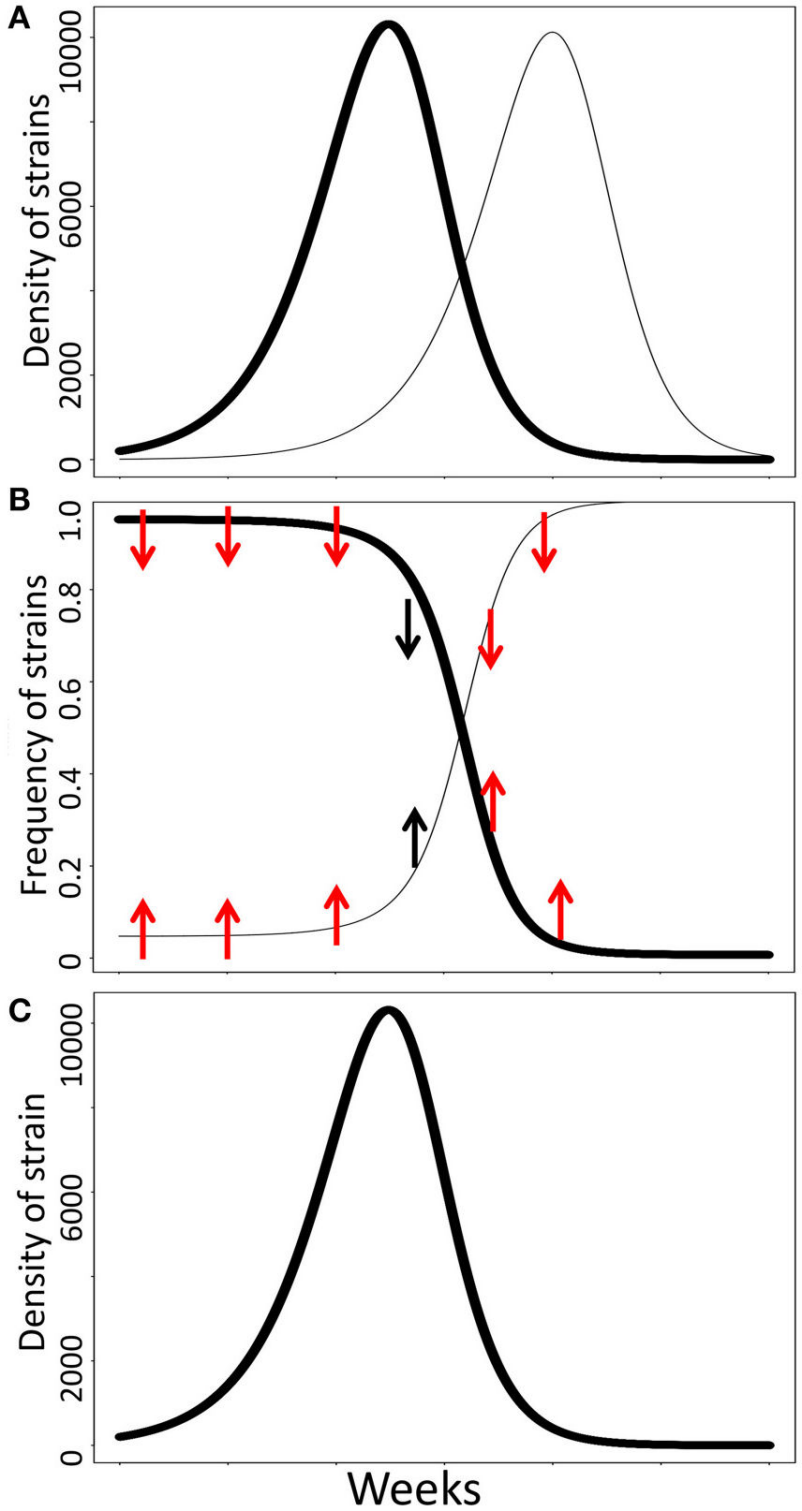
Influenza virus carrying rare HA or NA alleles do not have a selective advantage because they are relatively rare—a necessary condition of negative frequency-dependent selection—but because they are numerically rare compared to the number of susceptible hosts. **(A)** The population dynamics of two influenza strains (dark and light lines). Both strains increase numerically when they are numerically rare, but not relatively rare, and decrease after they become numerically common. Here, the maximal rate of increase of the first strain occurs prior to the second strain entering the population, despite remaining at the maximum relative abundance (100%). **(B)** The relative frequencies of the two influenza strains through time. If negative frequency-dependent selection were affecting the relative abundances of these strains, the common strain at time = 0 (dark line) should have lower fitness than the rare strain (light line). However, the numerical growth rate of the common strain remains high until it reduces the number of susceptible hosts, regardless of its frequency. Arrows indicate expected effects of negative frequency-dependent selection on the relative fitness of each strain given its relative abundance. Red arrows indicate the time periods when the expectations of negative frequency-dependent selection are not satisfied; black arrows indicate time periods when negative frequency-dependent selection expectations are satisfied. **(C)** The numerical growth rate and population dynamics of each strain have the same temporal patterns in the absence of alternative strains. Strain 1 remains at 100% frequencies throughout the time period, suggesting that relative abundance does not underlie changes in relative fitness.

The genetic diversity of haemagglutinin (HA) glycoproteins in the influenza virus is another conspicuous example of selection in a changing environment that is often confounded with negative frequency-dependent selection. The dynamics of HA alleles change over time such that rare alleles enter the population, rise to high population sizes, and subsequently decline toward extinction (Earn et al., 2002; Andreasen, 2003; Lin et al., 2003). The strains expressing a numerically common allele have relatively low fitness and decline in frequency because there are few hosts still susceptible to this strain, as hosts acquire immunity to strains with which they have been previously infected (Pease, 1987; Stegeman et al., 2004; Virseda et al., 2010). By contrast, strains expressing numerically rare alleles have many susceptible hosts available and enjoy high rates of secondary infections per infected host causing a numerical increase (Stegeman et al., 2004; Virseda et al., 2010). While there is undoubtedly strong selection at the HA locus, the selective advantage is derived not from relative rarity but from antigenic novelty (Plotkin and Dushoff, 2003; Nelson and Holmes, 2007; Cherry et al., 2009; Virseda et al., 2010), similar to Haldane’s example. The presence or frequency of alternative HA alleles does not affect the fitness (growth rate) or temporal dynamics of the alleles. That is, the population dynamics of a numerically rare allele is the same if the host population is already plagued by other numerically common strains (0.0001% when one novel allele enters a population of 106 infected hosts) and if it enters a host population in which no other influenza strain is circulating (100% when one novel allele enters a previous uninfected host population) (Figure 1). As the selective value of the allele is conditioned on the absolute abundance—but not the relative abundance—of the allele, it is unlikely that negative frequency-dependent selection is the evolutionary process underlying the polymorphism commonly observed at the HA locus. More likely, the common variant is changing its own environment such that there are few susceptible hosts in which new infections can establish, but it is not affecting the environment of alternative variants.

## DENSITY-DEPENDENT FITNESS DYNAMICS DESCRIBED AS NEGATIVE FREQUENCY-DEPENDENT SELECTION

A preeminent evolutionary biologist, Lewontin suggested that negative frequency-dependent selection should be pervasive because, whenever “a genotype is its own worst enemy, its fitness will decrease as it becomes more common” (Lewontin, 1974). As similar variants occupy similar ecological niches and are commonly their own worst enemy, this logic suggests that negative frequency-dependent selection should indeed be pervasive. However, “common” in this case refers not to relative abundance but absolute abundance. For example, the fitness (growth rate) of individuals within a monomorphic population, one in which the frequency of a genotype is always at 100%, decreases as it “becomes more common” in absolute abundance as it approaches a carrying capacity. Further, relatively rare variants suffer negative fitness effects in proportion to the absolute abundance of their numerically common competitors such that relative rarity may not provide a selective advantage.

There is an extensive literature describing fitness (growth rate) as a function of the absolute abundance of each variant in a population (Birch, 1955; MacArthur, 1962; MacArthur et al., 1967; Roughgarden, 1971; Emlen, 1985). The above scenario can be characterized using classical Logistic growth models that include competition among variants such that “a genotype is its own worst enemy” (Lotka-Volterra models) (Equation 1). The growth rates of the variants in these models are a function of the absolute abundance of each variant—discounted by their competitive abilities (*α_ij_*)—with respect to the carrying capacity (*K*), but are not explicitly conditioned on the abundance of the variants relative to each other. An interesting body of literature uses this modeling framework to describe the generation and maintenance of polymorphisms not through negative frequency-dependent selection mechanisms but through disruptive selection conditioned on the strength of competitive interactions and the abundance of each variant (ex Kisdi, 1999).

(1) $$\frac{dN_{1}}{dt}=r_{1}N_{1}\left(1-\frac{N_{1}+\alpha_{12}N_{2}}{K_{1}}\right)\;\frac{dN_{2}}{dt}=r_{2}N_{2}\left(1-\frac{N_{2}+\alpha_{21}N_{1}}{K_{2}}\right)$$

It is often challenging to distinguish the effect of numerical rarity from relatively rarity on the selective value of an allele through observations of patterns of allelic diversity. Experimental manipulations of the carrying capacity (*K*), potentially through resource supplementation, can assuage the reductions in relative fitness experienced by common variants that result from high densities without altering relative frequencies. In these experiments, the relative fitness of common variants should increase if the effects are associated with density while the relative fitness of the common and rare variants should not be altered if the allelic diversity is maintained by negative frequency-dependent selection.

## MULTIPLE NICHE POLYMORPHISMS DESCRIBED AS NEGATIVE FREQUENCY-DEPENDENT SELECTION

In the multiple niche selection model of balancing selection, the selective value of a trait is conditioned on its ability to exploit different environmental features in a heterogeneous habitat (Levene, 1953; Ravigne et al., 2004). Multi-niche selection maintains multiple variants in a population if each variant has a selective advantage in some available habitats while other variants are superior in other habitats. This idea—that environmentally variable selection can result in balanced polymorphisms—has a long history in the literature in which the foundational idea is stated by Dobzhansky (1982). Although incontrovertible examples of multi-niche selection maintaining polymorphism in natural populations are relatively rare, correct inference of the process resulting in balancing selection is necessary to generate hypotheses and design experiments to determine the ecological interactions or molecular mechanisms underlying the process.

The study of pattern, in isolation from the evolutionary processes that generated it, is not likely to advance general theories nor an understanding of specific systems (Cale et al., 1989). However, determining the processes responsible for balanced polymorphism patterns observed in nature is a difficult task (Barrett, 1988; Chaboudez and Burdon, 1995; Laine et al., 2011; Kazancioǧlu and Arnqvist, 2014). The balanced polymorphism at the outer surface protein *C* (*ospC*) locus in populations of *Borrelia burgdorferi*, the cause of human Lyme disease, provides a fitting example. Although the function of OspC remains unclear (Pal et al., 2004; Tilly et al., 2006, 2013; Xu et al., 2007; Onder et al., 2012; Carrasco et al., 2015), the within-population diversity at this locus bears all the hallmarks of balancing selection—large numbers of alleles in all local populations; allele frequencies that are more even than expected at neutrally evolving loci; and genetic evidence of an ancient polymorphism (Charlesworth et al., 1997; Qiu et al., 1997, 2002; May et al., 1999; Wang et al., 1999; Brisson and Dykhuizen, 2004).

Negative frequency-dependent selection and multi-niche selection have both been proposed as processes maintaining *ospC* polymorphisms, and both frameworks have empirical support (Qiu et al., 1997; Wang et al., 1999; Haven et al., 2011; Brisson et al., 2012; Seifert et al., 2015). The negative frequency-dependent selection model suggests that the polymorphism can be maintained if previously infected hosts are immune to subsequent infections by the same OspC variant but susceptible to novel variants, a molecular mechanism that has been demonstrated in laboratory animals (Gilmore et al., 1996; Probert et al., 1997; but see, Devevey et al., 2015). However, in this scenario the frequency or even presence of alternative OspC variants does not affect the number of susceptible hosts for the common strain, similar to the influenza example, arguing against negative frequency-dependent selection as an evolutionary process maintaining *ospC* polymorphisms. Further, negative frequency-dependent selection is most effective when few hosts remain susceptible to the common *ospC* variants, a pattern that is not observed in natural data sets (Brisson and Dykhuizen, 2004; Hanincova et al., 2006; Ogden et al., 2008; States et al., 2014; Vuong et al., 2014). Studies investigating allelic diversity at *ospC* from natural hosts consistently demonstrate that most natural reservoir hosts, those that are regularly infected with *B. burgdorferi*, are *rarely* infected with all of the common *ospC* variants (Brisson and Dykhuizen, 2004; Hanincova et al., 2006; Vuong et al., 2014; Mechai et al., 2016). Most hosts are, however, infected with a subset of the *ospC* variants, as expected if each host species represented a different ecological niche (Brisson and Dykhuizen, 2004; Hanincova et al., 2006; Vuong et al., 2014; Mechai et al., 2016). Further, host individuals of the same species, including humans, are often infected by the same subset of *ospC* variants across both time and geography (Seinost et al., 1999; Brisson and Dykhuizen, 2004; Hanincova et al., 2006; Dykhuizen et al., 2008; Wormser et al., 2008; Vuong et al., 2014; Mechai et al., 2016). The collective evidence suggests that the balanced *ospC* polymorphisms are more likely maintained by multi-niche selection—with each host species representing multiple niches (Brisson et al., 2011), one for each *ospC* variant by which it can be infected—than by negative frequency-dependent selection. These results suggest that the mechanisms causing the balanced polymorphism are more likely to involve *ospC* variant-by-host species interactions than to involve a memory immune response mechanism that is conserved across vertebrate species.

**FIGURE 2 ∣:**
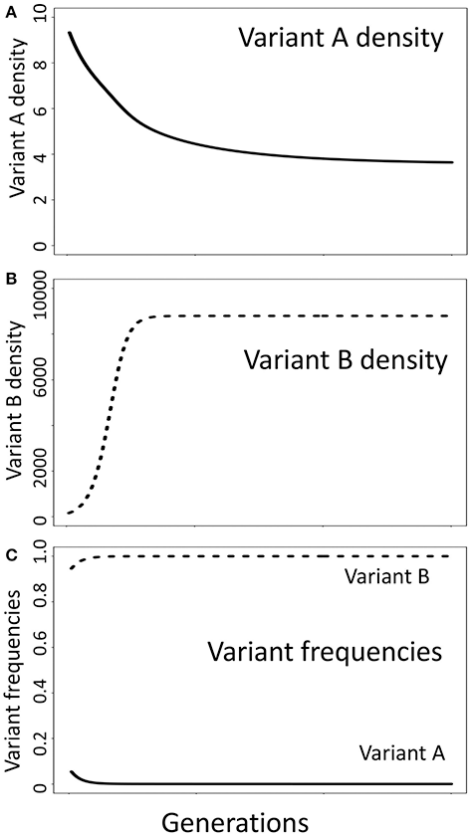
Multi-niche selection, an alternative model of balancing selection, does not require the core assumption of negative frequency-dependent selection models that relative fitness is a function of relative frequency in the population. Shown is a simulation where variant A has a selective advantage in niche A while variant B has a selective advantage in niche B (Appendix 1, Supplementary Material). Here, the carrying capacity in niche A is much lower than in niche B (K_A_ = 10, K_B_ = 10,000). At the start of the simulation, there are 10 variant A individuals (10% of the population) and 90 variant B individuals (90% of the population), yet the fitness (growth rate) of variant A individuals is much lower than for variant B individuals. This contradicts the expectations of negative frequency-dependent selection, where the frequency of variant A should increase as it is currently less frequent than variant B. Although the conditions necessary for negative frequency-dependent selection to maintain a stable polymorphism are not satisfied, both variants can be maintained in the population due to the selective advantage each enjoys in their preferred niche. Parameters used in the simulation: growth rate = 0.35, death rate in preferred niche = 0.05, death rate in non-preferred habitat = 0.25, migration among niches = 0.01.

It has been argued that “Selection in multiple niches is not an alternative to [negative] frequency-dependent selection… but a way of generating it” (Clarke, 1979). However, scenarios in which balanced polymorphisms can be maintained without a selective advantage favoring relatively rare variants are not uncommon, suggesting that these are two distinct evolutionary processes in at least some cases. To illustrate this point, image two variants occupying a heterogeneous habitat where each variant has a selective advantage in one niche but is disadvantaged in another, a classical multi-niche selection scenario (Levene, 1953; Ravigne et al., 2004). Here we assume that the carrying capacity in niche A is much lower than the carrying capacity in niche B (K_A_ = 10; K_B_ = 10^5^). In this scenario, variant B—which has a competitive advantage in niche B—can retain a fitness advantage (a greater *per capita* growth rate) even when it is more common than variant A—which has a competitive advantage in niche A. For example, in a population with 90 variant B individuals and 10 variant A individuals, variant B has a rapid *per capita* rate of increase while variant A does not increase (Figure 2). Here, the relatively common variant B has a “selective advantage” over the relatively rare variant A due to multi-niche selection, which is independent of negative frequency-dependent selection. Depending on the parameter values in this model, a balanced polymorphism can be maintained in the absence of rare advantage.

## COMMUNITY DIVERSITY DESCRIBED AS NEGATIVE FREQUENCY-DEPENDENT SELECTION

Prominent population geneticists including Williams and Maynard Smith, among many others, have demonstrated that the efficacy of natural selection decreases at increasing levels of biological organization such that selection among individuals within populations is much more efficient than selection among species within communities (Maynard Smith, 1964, 1976; Williams, 1966). Additionally, selection at higher levels of organization (i.e., among species within communities) “tends to be undermined by natural selection at lower levels” (i.e., among individuals with populations) (Wilson and Wilson, 2007). Nevertheless, several studies have suggested that negative frequency-dependent selection maintains species diversity within ecological communities. There is a rich empirical and theoretical history describing the causes and consequences of species diversity within ecological communities (Connell and Orias, 1964; Schoener, 1974; Lubchenco, 1978; Ricklefs and Schluter, 1993; Chesson, 2000; Wright, 2002). Mechanisms of coexistence function in two major ways: *equalizing* mechanisms minimize the average fitness differences between species while *stabilizing* mechanisms increase negative *intra*specific interactions relative to negative *inter*specific interactions (Chesson, 2000). Stabilizing mechanisms promote species coexistence and include mechanisms such as resource partitioning and frequency-dependent predation, as well as mechanisms that depend on spatial or temporal fluctuations in population densities or environmental factors. Equalizing mechanisms contribute to stable coexistence when they reduce large average fitness inequalities which might negate the effects of stabilizing mechanisms (Chesson, 2000). While some natural forces that affect the maintenance of community diversity have frequency-dependent mechanisms, this should not be mistaken for negative frequency-dependent selection which maintains polymorphisms within populations. Applying models of natural selection to levels of biological organization above the population level should be exercised only with the greatest of caution (Williams, 1966).

The Killing the Winner (KtW) hypothesis is a recent endeavor to understand patterns of diversity within communities using a negative frequency-dependent selection framework (Thingstad and Lignell, 1997; Thingstad, 2000). The KtW hypothesis suggests that predators target species that maximize reproductive effort over those that invest heavily in predator defense. Recent extensions of the KtW hypothesis suggest that this predator functional response promotes community diversity through negative frequency dependent selection. However, the functional response in this hypothesis is often not conditioned on the frequency of the prey species but on the presence or absence of character traits in the prey species (Thingstad and Lignell, 1997; Suttle, 2007; Winter et al., 2010; Koskella and Meaden, 2013). The “winner” in this hypothesis refers to species that invest resource into reproduction at the expense of investing in predator defenses, which may or may not correspond to the most frequent species (Winter et al., 2010). In these cases, neither the relative nor the absolute abundance of the prey species affects the functional responses of the predator.

## CONCLUDING REMARKS AND FUTURE PERSPECTIVES

Understanding the processes that produce or maintain diversity in natural populations is a central challenge in evolutionary biology. Negative frequency-dependent selection maintains many noted and striking polymorphisms in nature (Kojima and Tobari, 1969; Gigord et al., 2001; Charlesworth, 2006; Loisel et al., 2006; Fitzpatrick et al., 2007; Mitchell-Olds et al., 2007; Mokkonen et al., 2011), and many polymorphisms exist in the absence of a selective advantage favoring rare variants (Allison, 1954; Barton, 1986; Smith, 1990; Weatherall, 1997; Schmidt et al., 2000; Brisson and Dykhuizen, 2004). Ideally, one could unequivocally determine the causative process through observations of the patterns of variation in nature. Unfortunately, many processes result in identical patterns, especially when those patterns are observed over short time scales. In some cases, long-term observations of allelic dynamics can distinguish polymorphisms caused by mutation-selection balance or selection in a changing environment from a stable polymorphism resulting from balancing selection (Roy, 1998; Schmidt et al., 2000; Schmidt, 2001; Siemens and Roy, 2005; Olendorf et al., 2006; Koskella and Lively, 2009). Evidence suggesting negative frequency-dependent selection—such as allelic cycles where each allele gains a selective advantage as it becomes more rare—may also be observed from long-term observational studies (Gigord et al., 2001; Thrall et al., 2012). More directly, the patterns resulting from specific evolutionary processes can be tested through controlled and natural experiments such as manipulating allele frequencies in sub-populations (Roy, 1998; Schmidt et al., 2000; Olendorf et al., 2006; Koskella and Lively, 2009).

Ecological and molecular mechanisms are rarely deducible from patterns (Kershaw, 1963), but accurate identification of the evolutionary processes causing the pattern can generate hypotheses about these mechanisms. For example, the northern acorn barnacle, *Semibalanus balanoides*, shows clear evidence of a balanced polymorphism at the mannose-6-phosphate isomerase (*mpi*) locus (Hoffmann, 1981; McDonald, 1991). The pattern of *mpi* genotype frequencies among intertidal microhabitats, where one allele is common in high intertidal zones but rare in low intertidal zones, suggests that multi-niche selection maintains this polymorphism (Schmidt and Rand, 1999). Experimental manipulations of genotypes among microhabitats confirmed that multi-niche selection is the process responsible for the allelic variation (Schmidt et al., 2000; Schmidt and Rand, 2001). The molecular mechanism linking mannose utilization with survivorship in high intertidal zones, where temperature and desiccation stress is high, was subsequently elucidated through controlled laboratory experiments (Schmidt, 2001). As this and many other examples demonstrate, the ecological interaction or molecular mechanism underlying an evolutionary process can best be understood when the evolutionary process is accurately determined.

## Supplementary Material

Appendix 2 - Ordinary differential equation (ODE) approximations of the simulation in Appendix 1

Appendix 1 - Figure 2 data generated in R
