# Supplementary material for: Negative Frequency-Dependent Selection Is Frequently Confounding
Source: Front Ecol Evol. Author manuscript; Available in PMC 2021 Aug 12. (PMC8360343; doi:10.3389/fevo.2018.00010)
Supplement: Appendix 2 - Ordinary differential equation (ODE) approximations of the simulation in Appendix 1 [file NIHMS1727377-supplement-Appendix_2___Ordinary_differential_equation__ODE__approximations_of_the_simulation_in_Appendix_1.docx]

**Supplemental material**

**Figure S1 data generated in R**

### 3 genotypes S1S2, S1S3, S2S3

### S1 pollen can only pollinate S2S3 plant, 50% of time get S1S2, 50% S1S3

##Outline ###

## Start with Plants at different freq

## A pollen grain is chosen at random (by percent each is found in the population)

## pollen lands on 1 of the 100 plants (random number generator 1-100)

## Rejected if either allele of same plant is same as pollen

## if not rejected, makes seedlings with one of the 2 alleles (picked at random)

## repeat until 100 next generation plants

## repeat for 100 generations

##### PARAMETERS ####

S1S2<- 500 ## Starting pop of variant S1S2

S1S3<- 450 ## Starting pop of variant S1S3

S2S3 <- 50 ## Starting pop of variant S2S3

PopSize<-S1S2+S1S3+S2S3

S1<- (S1S2+S1S3)/(2*(S1S2+S1S3+S2S3)) ## starting number of S1 pollen grains

S2<- (S1S2+S2S3)/(2*(S1S2+S1S3+S2S3)) ## starting number of S2 pollen grains

S3<- (S2S3+S1S3)/(2*(S1S2+S1S3+S2S3)) ## starting number of S3 pollen grains

S1S2ng<-0

S1S3ng<-0

S2S3ng<-0

#### HOUSEKEEPING STUFF #####

generations<-5

pollens<-1000000

S1vector <- {}

S2vector <- {}

S3vector <- {}

S1vector[1] <- (S1S2+S1S3)/(2*(S1S2+S1S3+S2S3))

S2vector[1] <- (S1S2+S2S3)/(2*(S1S2+S1S3+S2S3))

S3vector[1] <- (S1S3+S2S3)/(2*(S1S2+S1S3+S2S3))

#### START OF MODEL SIMULATION #####

for (gens in 2:generations) { ## number of generations loop

pS1S2<-S1S2/(S1S2+S1S3+S2S3)

pS1S3<-S1S3/(S1S2+S1S3+S2S3)

pS2S3<-S2S3/(S1S2+S1S3+S2S3)

for (pol in 1:pollens) { ## mating loop

S1yes<-0

S2yes<-0

S3yes<-0

S1S2yes<-0

S2S3yes<-0

S1S3yes<-0

# choose pollen variant

pollenRand <-runif(1)

if (pollenRand<=S1) {S1yes<-1}

if (pollenRand > S1+S2) {S3yes<-1}

if (S1yes==0 & S3yes==0) {S2yes<-1}

# choose plant variant

PlantRand <-runif(1)

if (PlantRand<=pS1S2) {S1S2yes<-1}

if (PlantRand > pS1S2+pS1S3) {S2S3yes<-1}

if (S1S2yes==0 & S2S3yes==0) {S1S3yes<-1}

## next generation plants

if (S1yes == 1 & S2S3yes==1) {

alleleSelect<-runif(1)

if (alleleSelect<=.5){S1S2ng <- S1S2ng+1}

else {S1S3ng <- S1S3ng+1}

}

if (S2yes == 1 & S1S3yes==1) {

alleleSelect<-runif(1)

if (alleleSelect<=.5){S1S2ng <- S1S2ng+1}

else {S2S3ng <- S2S3ng+1}

}

if (S3yes == 1 & S1S2yes==1) {

alleleSelect<-runif(1)

if (alleleSelect<=.5){S1S3ng <- S1S3ng+1}

else {S2S3ng <- S2S3ng+1}

}

#Stop when 100 seedlings

if (S1S2ng + S1S3ng + S2S3ng ==PopSize){break}

} ## mating loop

S1S2<-S1S2ng

S1S3<-S1S3ng

S2S3<-S2S3ng

S1S2ng <-0

S1S3ng <-0

S2S3ng <-0

S1<- (S1S2+S1S3)/(2*(S1S2+S1S3+S2S3))

S2<- (S1S2+S2S3)/(2*(S1S2+S1S3+S2S3))

S3<- (S2S3+S1S3)/(2*(S1S2+S1S3+S2S3))

S1vector[gens] <- (S1S2+S1S3)/(2*(S1S2+S1S3+S2S3))

S2vector[gens] <- (S1S2+S2S3)/(2*(S1S2+S1S3+S2S3))

S3vector[gens] <- (S1S3+S2S3)/(2*(S1S2+S1S3+S2S3))

}## end generations

S1S2

S1S3

S2S3

S1vector

S2vector

S3vector

minVect<-{}

minVect[1]<-min(S1vector)

minVect[2]<-min(S2vector)

minVect[3]<-min(S3vector)

maxVect<-{}

maxVect[1]<-max(S1vector)

maxVect[2]<-max(S3vector)

maxVect[3]<-max(S3vector)

minTotal<-min(minVect)

maxTotal<-max(maxVect)

minTotal

maxTotal

plot(S1vector,type = "l", lwd=5, xlim=c(1, gens), ylim=c(minTotal-.01,maxTotal+.01), xaxt="n")

#axis(1, at = seq(1, 10, by = 1), las=2)

lines(S2vector,type = "l", lty=3, lwd=5)

lines(S3vector,type = "l", lty=4, lwd=5)

plot(S1vector,type = "l", lwd=5, xlim=c(1, gens), ylim=c(minTotal-.01,maxTotal+.01), xaxt="n")

axis(1, at = seq(1, 10, by = 1), las=2)

plot(S2vector,type = "l", lwd=5, xlim=c(1, gens), ylim=c(minTotal-.01,maxTotal+.01), xaxt="n")

axis(1, at = seq(1, 10, by = 1), las=2)

plot(S3vector,type = "l", lwd=5, xlim=c(1, gens), ylim=c(minTotal-.01,maxTotal+.01), xaxt="n")

axis(1, at = seq(1, 10, by = 1), las=2)

#plot(S2vector,type = "l", lty=3, lwd=5, xlim=c(1, gens), ylim=c(0,1))

#plot(dtvectorN1freq,type = "l", lwd=5, xlim=c(0, gen), ylim=c(0,1))

#plot(dtvectorN1,type = "n", xlim=c(1, gen), ylim=c(0,Ka))

#lo <- loess(dtvectorN1~time)

#xl <- seq(min(time),max(time), (max(time) - min(time))/1000)

#lines(xl, predict(lo,xl), col='black', lwd=5)

##plot(dtvectorN2,type = "n", xlim=c(1, gen), ylim=c(0,Kb))

#lo <- loess(dtvectorN2~time)

#xl <- seq(min(time),max(time), (max(time) - min(time))/1000)

#lines(xl, predict(lo,xl), col='black', lwd=5)

#plot(dtvectorN1freq,type = "n", xlim=c(0, gen), ylim=c(0,1))

#lo <- loess(dtvectorN1freq~time)

#xl <- seq(min(time),max(time), (max(time) - min(time))/1000)

#lines(xl, predict(lo,xl), col='black', lwd=5)

#plot(dtvectorN2freq,type = "n", xlim=c(0, gen), ylim=c(0,1))

#lo <- loess(dtvectorN2freq~time)

#xl <- seq(min(time),max(time), (max(time) - min(time))/1000)

#lines(xl, predict(lo,xl), col='black', lwd=5)

**Appendix 1 -. Figure 2 data generated in R**

### Make a model with 2 niches (A and B) and two variants (1 and 2)

### show how it is not frequency but abundance and carrying capacity that affect relative fitness

### cycle through differential fitness values (death rates in home vs away areas) and migration

###### values show when relative fitness changes and when polymorphism maintained

##### PARAMETERS ####

N1aStart<- 10 ## Starting pop of variant 1 (all start in their home niche)

N2bStart<- 100 ## Starting pop of variant 2 (all start in their home niche)

N1bStart <- 0 ## Starting pop of variant 1 in niche b

N2aStart <- 0 ## Starting pop of variant 2 in niche a

N1m <-0 ## migrant pool

N2m <-0 ## migrant pool

Ka<- 10 ## Carrying capacity of niche a

Kb<- 10000 ## Carrying capacity of niche b

rh <- .35 ### growth rate in correct niche

ra <-rh ## growth rate in incorrect niche (cycle through this in for loop)

## Birth rate same in both areas and controlled by K, death rate much higher in away (and happens first)

dh<-.05

##da - cycles

generations<-150

#### HOUSEKEEPING STUFF #####

#i <- seq(.1, .5, by=.01)

#j <- seq(0, .3, by=.01)

k <- seq(0, generations, by=1)

i <- seq(.25, .25, by=.01)

j <- seq(.01, .01, by=.01)

dtvectorN1<-{}

dtvectorN2<-{}

dtvectorN1freq<-{}

dtvectorN2freq<-{}

time<-{}

#### START OF MODEL SIMULATION #####

for (da in i) { ##Selection differential loop

for (m in j) { ## migration loop

N1a<- N1aStart ##resetting the starting population

N2b<- N2bStart

N1b<- N1bStart

N2a<- N2aStart

for (gen in k){## generations loop

#Deaths

N1a <- N1a * (1-dh) ## deaths in the home area (where fitness is higher)

N1b <- N1b * (1-da) ## deaths in the away area (where fitness is lower)

N2a <- N2a * (1-da)

N2b <- N2b * (1-dh)

#Births

N1a <- N1a * (1+rh* (1 - (N1a+N2a)/Ka))

N1b <- N1b * (1+ra* (1 - (N1b+N2b)/Kb))

N2a <- N2a * (1+ra* (1 - (N1a+N2a)/Ka))

N2b <- N2b * (1+rh* (1 - (N1b+N2b)/Kb))

#migration

N1aT<- N1a - N1a*m ##emigrants leaving the population

N1bT<- N1b - N1b*m

N2aT<- N2a - N2a*m

N2bT<- N2b - N2b*m

if (N1aT < 0) {N1aT<-0}

if (N2aT < 0) {N2aT<-0}

if (N1bT < 0) {N1bT<-0}

if (N2bT < 0) {N2bT<-0}

N1am <- m * (N1a + N1b)/2 ## immigrants joining a population

N1bm <- N1am

N2am <- m * (N2a + N2b)/2

N2bm<-N2am

NaSpace<- Ka-N1aT-N2aT ## migrants cannot displace the residents

NbSpace<- Kb-N1bT-N2bT## migrants cannot displace the residents

if(NaSpace<0){NaSpace<-0}

if(NbSpace<0){NbSpace<-0}

if ((N1am+N2am) > NaSpace){

N1a <- N1aT + NaSpace*N1am/(N1am+N2am)

N2a <- N2aT + NaSpace*N2am/(N1am+N2am)

} else {

N1a <- N1aT + N1am

N2a <- N2aT + N2am

}

if ((N1bm+N2bm) > NbSpace){

N1b <- N1bT + NbSpace*N1bm/(N1bm+N2bm) ## if the number of immigrants is too large, a proportion join the population

N2b <- N2bT + NbSpace*N2bm/(N1bm+N2bm)

} else {

N1b <- N1bT + N1bm

N2b <- N2bT + N2bm

}

N1<-N1a+N1b

N2<-N2a+N2b

dtvectorN1[gen]<-N1

dtvectorN2[gen]<-N2

dtvectorN1freq[gen]<-N1/(N1+N2)

dtvectorN2freq[gen]<-N2/(N1+N2)

time[gen]<-gen

} ## end generations loop

} ##end migration loop

}## end selection loop

**Appendix 2 – Ordinary differential equation (ODE) approximations of the simulation in Appendix 1**

In the multiple niche selection model of balancing selection, the selective value of an allele is conditioned on its ability to exploit different environmental features in a heterogeneous habitat [59,60]. This simple ODE model is illustrative of a multiple niche polymorphisms that can maintain a stable polymorphism without selection favoring relatively rare variants.

$$\frac{{dN}^{1}}{dt}= \frac{{dN}_{A}^{1}}{dt}+\frac{{dN}_{B}^{1}}{dt}$$

$$\frac{{dN}^{2}}{dt}= \frac{{dN}_{A}^{2}}{dt}+\frac{{dN}_{B}^{2}}{dt}$$

$$\frac{{dN}_{A}^{1}}{dt}= N_{A}^{1}\left( r\left( 1-\frac{N_{A}^{1}+N_{A}^{2}}{K_{A}} \right) \right)- N_{A}^{1}\left( m+d_{h} \right)+ N_{B}^{1}m$$

$$\frac{{dN}_{B}^{1}}{dt}= N_{B}^{1}\left( r\left( 1-\frac{N_{B}^{1}+N_{B}^{2}}{K_{B}} \right) \right)- N_{B}^{1}\left( m+d_{a} \right)+ N_{A}^{1}m$$

$$\frac{{dN}_{A}^{2}}{dt}= N_{A}^{2}\left( r\left( 1-\frac{N_{A}^{1}+N_{A}^{2}}{K_{A}} \right) \right)- N_{A}^{2}\left( m+d_{h} \right)+ N_{B}^{2}m$$

$$\frac{{dN}_{B}^{2}}{dt}= N_{B}^{2}\left( r\left( 1-\frac{N_{B}^{1}+N_{B}^{2}}{K_{B}} \right) \right)- N_{B}^{2}\left( m+d_{a} \right)+ N_{A}^{2}m$$

where $N^{1}$is the total abundance of variant 1, $N_{A}^{1}$ is the abundance of variant 1 in habitat $A$ (its preferred habitat), $K_{A}$ is the carrying capacity of habitat $A$, $d_{h}$ and $d_{a}$ are the death rates in the preferred (*home*) and non-preferred (*away*) habitats, and $r$ and $m$ are the intrinsic growth and migration rates (which do not differ among variants). Substituting parameter values similar to those used in the simulation ($r=0.35;$ $K_{A}=100$; $K_{B}=1000$; $d_{h}=0.05$; $d_{a}=0.25$; $m=0.01$), a stable equilibrium can be found at $N^{1}=45$ and $N^{2}=869$ ($p=0.049$; $q=0.951$). The derivatives around $N^{1}=100$; $N^{2}=500$ ($p=0.167$; $q=0.833$) are negative for $N^{1} (\frac{{dN}^{1}}{dt}=-5$) and strongly positive for $N^{2} (\frac{{dN}^{2}}{dt}=63.5)$, which is inconsistent with the expectation under negative frequency-dependent selection in which the rare variant should increase in frequency due to its rare advantage. This ODE model is qualitatively similar to one published in Ravigne *et al* (details in Appendix 1 of [60]):

$$\Delta p=pq\frac{c_{1}\left( h_{A,1}w_{1}-h_{a,1}v_{1} \right)\left( ph_{A,2}w_{2}+qh_{a,2}v_{2} \right)-c_{2}\left( h_{A,2}w_{2}+h_{a,2}v_{2} \right)\left( ph_{A,1}w_{1}+qh_{a,1}v_{1} \right)}{\left( ph_{A,1}w_{1}+qh_{a,1}v_{1} \right)\left( ph_{A,2}w_{2}+qh_{a,2}v_{2} \right)}$$

where $c_{1}$ is the proportion of habitat 1, $w_{1}$ is the viability of $A$ in habitat 1 (where $A$ has a survival advantage), $v_{1}$ is the viability of $a$ in habitat 1, and $h_{A,1}$ is the habitat preference of $A$ for habitat 1. Substituting parameter values similar to those used above ($c_{1}=0.99; c_{2}=0.01; h_{A,1}=h_{a,2}=0.9999; h_{a,1}=h_{A,2}=0.0001; w_{1}=v_{2}=5; w_{2}=v_{1}=1$), a stable equilibrium can be found at $p=0.99$ and $q=0.01$. The derivative around $p=0.9$ are nevertheless positive for $p (\Delta p=0.09$), despite being at a much higher frequency, suggesting that the relative selective value of $p$ does not become negative due to an increase in frequency.
