# Supplementary material for: Negative Frequency-Dependent Selection Is Frequently Confounding
Source: Front Ecol Evol. Author manuscript; Available in PMC 2021 Aug 12. (PMC8360343; doi:10.3389/fevo.2018.00010)
Supplement: Appendix 1 - Figure 2 data generated in R [file NIHMS1727377-supplement-Appendix_1_-_Figure_2_data_generated_in_R.docx]

**Figure S1.** The classical self-incompatibility allele model [19] represented with three *S-*alleles. **A.** *S_1_S_1_* homozygote plants produce pollen containing the *S_1_* allele and can be pollinated only by pollen containing an *S_2_*- or *S_3_*-allele. Similarly, *S_2_S_2_* homozygotes produce *S_2_*-containing pollen and are pollinated by *S_1_*- or *S_3_*-pollen. Heterozygote plants produce pollen with either of its two alleles but cannot be pollinated by either pollen variant. Alleles that are relatively rare in the population have a selective advantage over common alleles as rare-allele pollen are much more likely to pollinate a receptive ovule. In contrast, common-allele pollen are likely to attempt to pollinate a plant containing that common allele and be rejected, resulting in limited reproductive success. **B.** The temporal dynamics of alleles in this system are likely to fluctuate as expected when rare alleles have a selective advantage. Here, when 81% of the plants are homozygous *S_1_S_1_* (time 0), the *S_2_*-containing pollen (~10%) has a high probability of finding an *S_1_S_1_* plant and successfully breeding. By contrast, the *S_1_*-containing pollen is highly unlikely to find a *S_2_S_2_* homozygote (~1% of all plants), resulting in very low breeding success. Due to the limited breeding success, the *S_1_* allele will decrease in frequency until *S_1_*-containing pollen is rare and thus becomes more likely to find a receptive mate. These dynamics occur because pollen grains with common alleles are limited in terms of mates, while pollen grains with rare alleles are not. Hence, plants with rare alleles have a selective advantage in terms of mating.
